# Intravenous Infusion of Monocytes Isolated from 2-Week-Old Mice Enhances Clearance of Beta-Amyloid Plaques in an Alzheimer Mouse Model

**DOI:** 10.1371/journal.pone.0121930

**Published:** 2015-04-01

**Authors:** Lindsay A. Hohsfield, Christian Humpel

**Affiliations:** Laboratory of Psychiatry and Experimental Alzheimer’s Research, Department of Psychiatry and Psychotherapy, Medical University of Innsbruck, Innsbruck, Tyrol, Austria; Nathan Kline Institute and New York University School of Medicine, UNITED STATES

## Abstract

Alzheimer’s disease (AD) is characterized by the deposition of β-amyloid (Aβ) senile plaques and tau-associated neurofibrillary tangles. Other disease features include neuroinflammation and cholinergic neurodegeneration, indicating their possible importance in disease propagation. Recent studies have shown that monocytic cells can migrate into the AD brain toward Aβ plaques and reduce plaque burden. The purpose of this study was to evaluate whether the administration of intravenous infusions of ‘young’ CD11b-positive (+) monocytes into an AD mouse model can enhance Aβ plaque clearance and attenuate cognitive deficits. Peripheral monocytes were isolated from two-week-old wildtype mice using the Pluriselect CD11b+ isolation method and characterized by FACS analysis for surface marker expression and effective phagocytosis of 1 μm fluorescent microspheres, FITC-Dextran or FITC-Aβ_1–42_. The isolated monocytes were infused via the tail vein into a transgenic AD mouse model, which expresses the Swedish, Dutch/Iowa APP mutations (APPSwDI). The infusions began when animals reached 5 months of age, when little plaque deposition is apparent and were repeated again at 6 and 7 months of age. At 8 months of age, brains were analyzed for Aβ+ plaques, inflammatory processes and microglial (Iba1) activation. Our data show that infusions of two-week-old CD11b+ monocytes into adult APPSwDI mice results in a transient improvement of memory function, a reduction (30%) in Aβ plaque load and significantly in small (<20 μm) and large (>40 μm) plaques. In addition, we observe a reduction in Iba1+ cells, as well as no marked elevations in cytokine levels or other indicators of inflammation. Taken together, our findings indicate that young CD11b+ monocytes may serve as therapeutic candidates for improved Aβ clearance in AD.

## INTRODUCTION

Alzheimer’s disease (AD) is a progressive neurodegenerative disease characterized by the deposition of tau-associated neurofibrillary tangles and senile plaques, composed of β-amyloid (Aβ), activated microglia, reactive astrocytes, and dystrophic neurons and synapses [1]. AD symptoms first appear as short-term lapses in memory, however, further progress into significant deficits in cognitive and executive functions. Although amyloid plaque deposition does not correlate to cognitive decline, accumulating evidence indicates that soluble Aβ oligomers may play a detrimental role in AD pathology and associated cognitive impairment [2,3]. Therefore, several AD therapeutic strategies have focused on developing methods that reduce Aβ accumulation or enhance Aβ clearance in the central nervous system (CNS).

Convincing evidence indicates that the infiltration of monocyte-derived cells (i.e. monocytes and macrophages) from the periphery into the CNS and perivascular spaces may help restrict amyloid deposition and prevent cognitive decline [4–6]. These studies have shown that bone marrow-derived cells or monocytes are recruited to the brain (triggered by Aβ_40_ or Aβ_42_) and can restrict amyloid plaque deposition in AD transgenic mice [7,8]. In addition, a recent study has shown that patrolling monocytes are naturally attracted to and can eliminate Aβ within the lumen of veins [6]. Furthermore, studies have shown that restricting peripheral monocyte migration into the brain (via CCR2 deficiency) of AD transgenic mice impairs microglial accumulation and accelerates disease progression [9]. CCR2 deficiency, specifically in bone marrow-derived cells, in AD transgenic mice enhances Aβ pathology and aggravates cognitive impairment [10,11]. In fact, promoting peripheral macrophage infiltration attenuates amyloid deposition and improves cognitive function [5,10,12–16]. Taken together, these findings indicate that the administration and subsequent recruitment of peripheral monocytic cells into the brain may have important implications for future AD therapies.

Increasing evidence indicates that monocyte-derived cells are more effective phagocytes for Aβ than microglia [4,17,18]. In fact, a recent study has shown that peripheral macrophages are more efficient at degrading oligomerized tau than microglial cells [19]. However, others report that macrophages from AD patients are incapable of degrading Aβ [20]. It could be possible that with age or disease onset, the cells of the immune system, including monocytes and macrophages, undergo changes leading to compromised function and inability to clear AD pathology. Previous investigations have shown that monocytes isolated from the elderly exhibit altered monocyte numbers and subsets, altered phenotypes (i.e. increased or decreased expression of cell surface markers), altered systemic and intracellular cytokine levels, and impaired phagocytosis [21–25]. We hypothesize that ‘younger’ monocytes have a better functional capacity, including survival, stem cell-like potential, and phagocytosis and thus, could aid in resolving AD pathology. Interestingly, a recent study demonstrated that the exposure of aged animals to young blood results in improved cognitive function including spatial learning and memory [26]. Thus, in this study we sought to investigate the role of ‘young’ peripheral monocytes in amyloid burden and cognitive function.

In the present study, we investigated the effects of CD11b+ monocytes (isolated from two-week-old wildtype mice) infused into the APPSwDI AD transgenic mouse model. Here, we evaluated cognition (learning) and anxiety, as well as Aβ plaque load, microglia activation and inflammatory marker expression in the cortex. Our findings indicate that infusion of ‘young’ peripheral monocytes results in the reduction of plaques and microglial cells in the cortex of APPSwDI animals, providing evidence in favor of the use of monocytes as therapeutic strategies against AD.

## METHODS

### Animals

APPSwDI transgenic mice (C57BL/6-Tg(Thy1-APPSwDutIowa) BWevn/ Mmjax; The Jackson Laboratory), expressing amyloid precursor protein (APP) harboring the Swedish K670N/M671L, Dutch E693Q, and Iowa D694N mutations, were housed at the Innsbruck Medical University animal facility providing open access to food and water under 12 h/12 h light-dark cycles. These mice were generated and have been extensively characterized previously by Davis et al. [27]. All animals were genotyped according to standardized methods. All animal experiments were approved by the Austrian Ministry of Science and Research (BMWF-66.011/0044-II/3b/2011 and BMWF-66.011/0059-II/3b/2011) and conformed to the Austrian guidelines on animal welfare and experimentation. All possible steps were taken to reduce suffering and the number of animals used during the experiment.

### Experimental set-up

**Fig 1:**
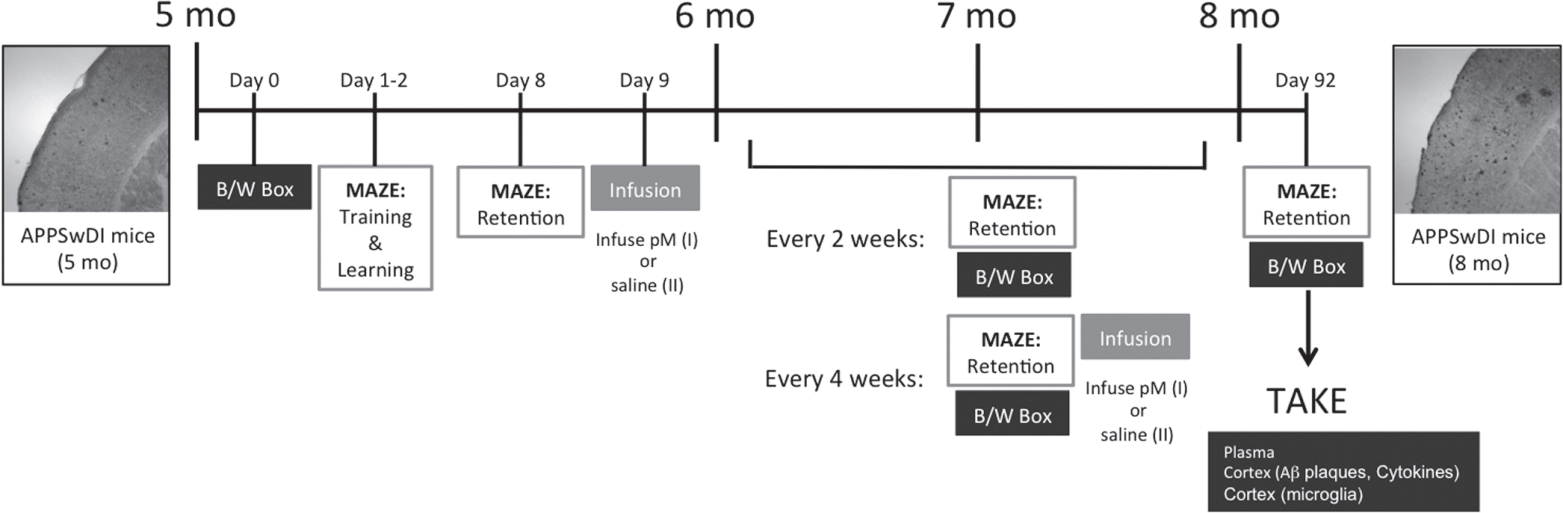
Experimental set-up for monocyte infusions, cognitive evaluations and neurobiological analysis. At 5 months of age (mice do not display any extracellular plaques), APPSwDI mice were evaluated by the black/white (B/W) box and 8-arm radial maze, which included both training, learning and retention sessions. Following these evaluations, mice were infused intravenously with (I) approx. 5 x 10^6^ young CD11b+ monocytes (isolated from 14 day-old wildtype mice; n = 8) or with (II) saline (n = 7). Every two weeks, animals were tested in the B/W box and 8-arm maze for changes in anxiety and memory, respectively. Intravenous infusions were repeated at months 6 and 7, following cognitive evaluations. At 8 months of age, mice were sacrificed (TAKE) following retention and analyzed.

The experiment (**Fig. 1**) began when APPSwDI animals reached 5 months of age. To establish baseline fear and cognitive function, animals were tested in the black/white box (day 0) and 8-arm radial maze (training on day 1; learning sessions on day 2) as described below. On day 8, animals were placed again in the maze to evaluate retention (memory). On the following day, animals were infused with monocytes. Two weeks later, mice (6-month-old) were tested again for retention and brought back to the animal department. Following another two weeks, animals were again tested for retention and on the subsequent day infused with monocytes. This was again repeated at 7 months of age. At 8 months of age, retention was tested again prior to sacrifice (take).

### Black/white test box

Anxiety was assessed using the black/white (B/W) box, also known as the light/dark exploration test, developed by Crawley [28,29]. This model consists of two inter-connected compartments that vary in color (white/black) and illumination (light/dark). Animals were tested over 5 sessions; each session lasting 5 min long and 30 min apart. During the test, mice are placed into the white, illuminated area where under control conditions they will rapidly move into the dark area due to their innate aversion to brightly lit areas. To quantify anxiety, different measures were recorded: time in the white area (%, sec), time in the black area (%, sec), the first time leaving white to black area (sec), the number of transitions (white to black area), and the time in each quadrant (Q1-Q9) in the white area.

### Eight-arm radial maze

Spatial learning and memory was assessed using a modified win-shift procedure in an 8-arm radial maze (PanLab, Spain) previously described by us [30, 31]. The maze consists of a central starting arena from which eight identical Plexiglas arms radiate. Cups were placed at the ends of each arm where water could be placed as a reward (bait). To facilitate spatial navigation, small high contrast visual cues were placed above the entrance to four arms. To exclude olfactory interference, the maze apparatus was cleaned with 70% ethanol following each trial. The behavioral test equipment was automatically controlled and monitored by a computer equipped with Mazesoft 8.1.9 Software. In order to acclimatize animals to the apparatus and experimental set-up, animals were given 4 training sessions on day 1 and then placed on water restriction overnight to increase motivation for finding water (baits). On day 2, prior to behavioral testing, all arms were baited with 30 μl of water and mice were given 5 min to explore the maze. Spatial learning was tested on day 2 with eight consecutive sessions. Following this, animals were placed back on normal water consumption. For retention (memory) trials, animals were placed on water restriction overnight on day 7 and then tested the following day (day 8) with two sessions (at least 1 h apart). Quantification of cognitive performance (spatial learning and memory) was done by calculating the number of working memory errors made and the entries to repeat measure. Working memory errors are defined as errors that occur when an animal re-enters an arm that has already been visited. The entries to repeat measure captures choice accuracy and is defined by the number of correct entries into baited arms until an error is made.

### Isolation of peripheral blood mouse CD11b-positive monocytes

Six-month-old adult mice or 2 week old young mice (C57BL/6N; Charles River, Germany) were given an intraperitoneal overdose of sodium thiopental (12.5 mg; Sandoz, Austria) and perfused with 20 ml of 10 mM phosphate-buffer saline (PBS)/2.7 mM (5.5 mM) EDTA/25 mg/ml heparin, pH 7.3 through the left ventricle. The collected effluent was centrifuged at 550×g for 10 min at 4°C. The cell pellet was then resuspended in 4 ml of PBS/EDTA solution and 380 μl (40 μl /1 x 10^6^ target cells) of S-pluriBead suspension (pluriBead S-Bead CD11b Cell Separation KIT, pluriSelect) was added and incubated for 30 min on the provided pluriSelect pluriPlix at ~10 rpm/7.5° angle at room temperature. Following the incubation, the cell suspension was poured directly onto the strainer and then washed 14x with 1 ml of wash buffer in a circular motion. Following attachment of the provided connector, tube and strainer, 1 ml of detachment buffer was carefully added to the strainer (containing the isolated CD11b target cells) and the cells were then incubated for 10 min at room temperature. Following incubation, 1 ml of wash buffer was added to the strainer and cells were separated from the beads by pipetting up and down (10x). The Luer-Lock was opened and 1 ml of wash buffer was added to allow detached CD11b+ cells to run into the provided tube. The strainer was then washed 10x with 1 ml of wash buffer. The cells were then centrifuged at 250×g for 10 min. The supernatant was carefully discarded and cells were resuspended in 100 μl of desired vehicle (e.g. FACS or infusion buffer). Approximately 10.7 ± 0.8 million (n = 8) cells were isolated from one animal.

### Evaluation of phagocytosis by mouse CD11b-positive monocytes

The phagocytic activity of monocytes was assessed using FITC-Dextran (Sigma Aldrich, 100 ng), FluoSpheres Red (580/605) Fluorescent Microspheres (Molecular Probes, 1 μm, 3.6 x 10^7^ microspheres/ml), and FITC-β-Ala-Amyloid β-Protein (1–42) (Bachem, 100 ng). Approximately 500,000 cells were resuspended in 500 μl of culture medium (MEM + 1 mg/ml BSA + 0.35 mg/ml NaHCO_3_, pH 7.2) ± 1 μg/ml lipopolysaccharide (LPS) and incubated overnight at 37°C/5% CO_2_, then centrifuged (300xg 10 min), resuspended in 100 μl FACS flow and analysed (BD FACS Calibur).

Cells were also characterized for their antigen expression. Following cultivation, cells were centrifuged at 300×g for 10 min, resuspended in 50 μl of FACS buffer (1% EDTA, 0.5% FCS, pH 7.1) containing primary antibodies against CD11b (1:25; BD, 557395), CD11c (1:25; Miltenyi, 130-091-842), CD14 (1:25; BD, 553739), CD45 (1:25; Miltenyi, 130-091-609), CD68 (1:5; Thermo Fisher Scientific, MA1-82739), F4/80 (1:10; Serotec/Biorad, MCA497FT), Ly6C (1:25; Miltenyi, 130-093-134), and major histocompatibility complex II (MHCII; 1:25, Miltenyi, 130-081-601) and incubated at 4°C for 30 min. Cells were subsequently washed, centrifuged and resuspended in 100 μl of FACS Flow and analyzed. All necessary IgG (IgG2a, 2b(k) and IgG1) controls were included.

### Intravenous infusion of monocytes

Following behavioral testing, male APPSwDI mice received an intravenous (i.v.) injection via the lateral tail vein of ~5 x 10^6^ CD11b-positive monocytes (obtained from 2 week old wildtype mice) in 100 μl of heparinized saline at five, six, and seven months of age. Local anesthetic (5% Emla, AstraZeneca) was applied to dampen pain prior to injection. Animals receiving saline alone served as negative controls.

### Plasma and tissue collection

At the end of the experiment, animals were anesthetized by subcutaneous sodium thiopental (12.5 mg/ml, 1 ml) injection. Blood was taken directly from the heart, collected in EDTA tubes, and centrifuged at 400 ×g for 10 min. Plasma was stored at -80°C until further use. The brain was removed and a medial sagittal cut was made to divide the brain into two hemispheres. The left hemisphere was post-fixed in 4% PFA overnight and then stored in a 20% sucrose/PBS solution until further use for immunostaining. Regions of the cortex in the right hemisphere were removed and immediately frozen at -80°C (inflammatory markers and Western Blots).

### Preparation of cortical extracts

Cortical tissue was thawed and dissolved in 100 μl ice-cold PBS containing a protease inhibitor cocktail (P-8340, Sigma), homogenized using an ultrasonic device (Hielscher Ultrasonic Processor, Germany) and then centrifuged at 16000 ×g for 10 min at 4°C. The supernatant was collected and samples were stored at -80°C until further use (ELISA and Western Blots). Total protein was determined by Bradford protein assay.

### Inflammatory markers ELISA analysis

The detection of inflammatory proteins (monocyte chemotactic protein-1, MCP-1; macrophage inflammatory protein-2, MIP-2; tumor necrosis factor-α, TNF-α; interleukin-1β, IL-1β) was performed using the Thermo Scientific SearchLight Protein Array Technology (THP Medical Products, Vienna) as previously described by us [32]. Briefly, cell extracts (diluted 1:2 in diluent) or calibrated standards were added to coated wells of the provided plate and incubated for 3 h. After washing, the biotinylated antibodies were added and following 30 min incubation the wells were washed again and incubated with streptavidin-horseradish peroxidase conjugate. After the final washing step the SuperSignal Chemiluminescent Substrate was added. All incubation steps were carried out on a shaker at 20°C. The luminescent signal was detected using a compatible CCD imaging and analysis system and the absorbance was measured at 450 nm. The concentration of each sample was quantified by comparing spot intensities with the corresponding standard curves calculated from values of the standard samples using the SearchLight Array Analyst Software.

### Western Blot

Western blot analysis was performed as previously described by us [32]. Following extraction, cells were centrifuged and 20 μl of supernatant was loaded with sample buffer. Samples were separated in 10% Bis-Tris SDS-polyacrylamide gels for 35 min at 200V and then electrotransferred to nylon-PVDF Immobilon-P^SQ^ membranes for 90 min at 30V in 20% methanol blotting buffer. The Western Breeze Chromogenic System was used for the detection of specific proteins in cortical extracts. Briefly, blots were blocked for 30 min in blocking buffer, incubated with primary antibodies against actin (1:1000; Sigma, A2066), amyloid precursor protein (APP; 1:1000; Abcam, ab32136), catalase (1:10,000; Thermo, PA1-28372), or matrix metallopeptidase 2 (MMP-2; 1:1000; Abcam, ab37150) for 90 min, washed, and then incubated in alkaline phosphatase conjugated anti-rabbit (or anti-goat) IgG for 30 min. After washing, bound antibodies were visualized by p-nitro blue tetrazolium chloride and 5-bromo-4-chloro-3-indolyl phosphate. Values for protein expression were obtained by quantifying optical density of protein bands (corrected for actin) using Image J software.

### Immunohistochemistry

Immunohistochemistry was performed as previously described by us [30,31,33]. Following fixation, the left brain hemisphere was placed on a cork, frozen in a CO_2_ stream and subsequently cut into 40-μm cryostat (Leica CM 1950) sections. Monocytes were cultivated on collagen-coated (concentration, company) Nunc Lab-Tek II chamber slides (Thermo Scientific) and following incubation fixed with 4% PFA for 30 min at room temperature. The brain sections or cells were then washed with PBS and incubated in PBS/0.1% Triton (T-PBS) for 30 min at 20°C while shaking. To quench endogenous peroxidase, sections/cells were treated with PBS/1%H_2_O_2_/5% methanol. After incubation, the sections/cells were then blocked in T-PBS/20% horse serum (GIBCO Invitrogen)/0.2% BSA (SERVA) for 30 min at 20°C shaking. Following blocking, brain sections/cells were incubated with Aβ [4–5 kD, Invitrogen, 1:200], Aβ [1–16, Covance, 1:1000], or ionized calcium-binding adapter molecule 1 (Iba1) in T-PBS/0.2% BSA overnight at 20°C. Cells were then incubated with DAPI (1:10,000, Sigma) and propidium iodide (PI, 1–2 μg/ml, Sigma) for 1 h in the dark (shaking) at room temperature. Slides were then washed, cover-slipped with Vectashield Mounting Medium (Vector Laboratories), and visualized under a fluorescent microscope. In the case of non-conjugated primary antibodies, sections were then washed and incubated with the corresponding biotinylated secondary antibody (1:200, Vector Laboratories) in T-PBS/0.2% BSA for 1 h at 20°C shaking. Following secondary antibody incubation, sections were rinsed with PBS and incubated in avidin-biotin complex solution (Elite ABC kit, Vector Laboratories) for 1 h at 20°C shaking. Finally, the sections were washed with 50 mM Tris-buffered saline (TBS) and then incubated in 0.5 mg/ml 3,3’-diaminobenzidine (DAB, Sigma)/TBS/0.003% H_2_O_2_ at 20°C in the dark until a signal was detected. Once DAB staining was visible, the reaction was stopped by adding TBS to the sections. The brain sections were rinsed with TBS, mounted onto glass slides, cover-slipped with Entellan (Merck, Darmstadt, Germany), and then evaluated under the microscope by a blind observer.

### Microscopic evaluations

Images were captured with an Olympus BX61 (ProgRes C14 camera) microscope using Openlab 5.5.0 imaging software and acquired under the same exposure settings. For quantification, two to four brain sections per animal were evaluated for cortical staining patterns under the 10x (Iba1) or 20x (Aβ) objectives. The number of Aβ-positive plaques and Iba1-positive cells were evaluated and quantified using ImageJ software (NIH). Images were normalized to the same threshold levels and converted into binary formats. Iba1-positive cells were quantified using the particle analysis tool, set at 35–1000 μm^2^ in a 3.8 mm^2^ area. Plaque burden was calculated using area occupied by plaques divided by total cortical region. For specific plaques sizes, plaques were counted between 25–400 μm^2^, 400–1600 μm^2^, and >1600 μm^2^ within a 1.9 mm^2^ area. Evaluations were carried out by a blinded investigator.

### Statistical analysis

All data are reported as mean ± SEM (n = independent experiments or individual animals). Differences between mean values were determined using the Student’s t-test or a one-way ANOVA with a Fisher least significant difference post hoc test. P values < 0.05 were considered significant.

## RESULTS

### Characterization of CD11b-positive peripheral blood mouse monocytes

**Fig 2:**
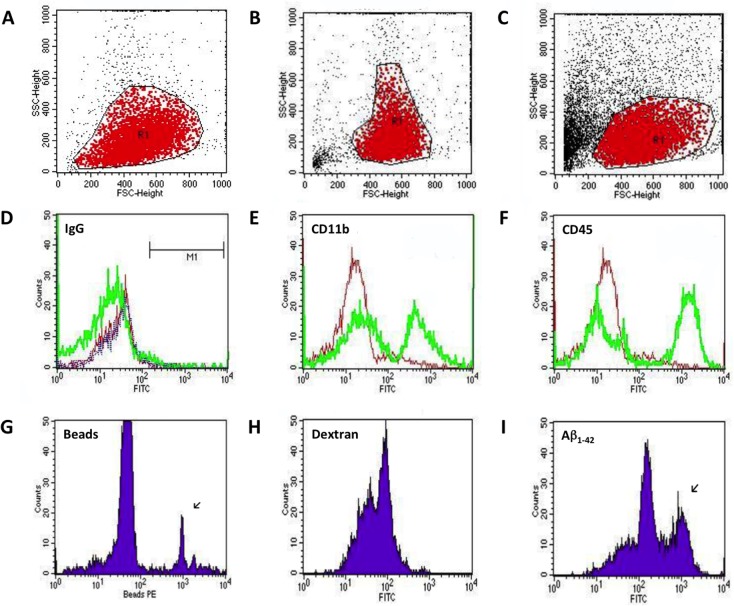
FACS analysis of CD11b-positive peripheral blood mouse monocytes. FACS analysis was performed on peripheral blood mouse monocytes isolated from young (14 days; **A**; n = 13) or adult (6 month, **B**; n = 9) C57BL/6N mice following CD11b pluriBead (Pluriselect) cell separation. CD11b-positive cells were evaluated immediately following isolation (**A&B**; n = 9) or following 24 h incubation (**C-I**; n = 9). Cells were evaluated for surface marker expression of CD11b and CD45 (**E&F**, green) as well as their ability to phagocyte 1 μm fluorescent microspheres (**G**; n = 10), or FITC-Dextran (**H**; n = 11) or FITC-Aβ_1–42_ (**I**; n = 13). IgG controls (IgG2a and IgG1) were used as a negative control (**D**).

Peripheral blood mouse monocytes were collected from the periphery of young (2 weeks; **Fig. 2A**) and adult (6 months, **Fig. 2B**) mice and subsequently evaluated for surface marker expression and functional properties (i.e. response to LPS, phagocytosis abilities). Following CD11b pluriBead cell separation and isolation, cells appeared to lose all surface marker expression as measured by FACS analysis (data not shown), however, regained this expression over time. Following 24 h incubation in the presence or absence (**Fig. 2C**) of LPS, cell surface marker expression for CD11b and CD45, common markers for monocyte-derived cells, became apparent (**Fig. 2E & 2F**), compared to respective IgG controls (**Fig. 2D**).

**Fig 3:**
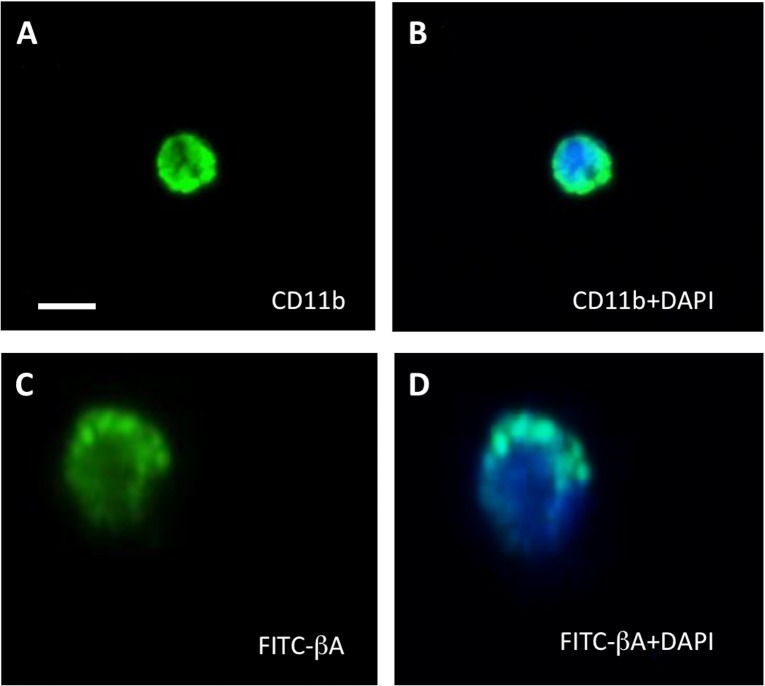
Microscopy analysis of CD11b immunostaining and phagocytosis of FITC-beta-amyloid (Aβ). Following 24 h incubation in the absence (**A&B**) or presence (C&D) of FITC-Aβ, CD11b-positive cells (isolated from adult C57BL/6N mice) were evaluated for CD11b surface marker staining (**A**) and engulfment of FITC-Aβ_1–42_ (**C**). Images illustrate apparent fluorescent staining (green) in healthy cells stained positive for nuclear (blue) DAPI (**B&D**). Cells displayed diffuse cytoplasmic granulated/ punctate staining of phagocytosed FITC-Aβ_1–42:_ (**C&D**). Scale bar = 10 μm (A&B), 5 μm (C&D).

The expression of CD11b was also verified by immunofluorescence stainings (**Fig. 3A & 3B**). CD68 was also detected in approximately 24% of monocytes following 24 h incubation without LPS and 32% with LPS stimulation. F4/80 was detected in approximately 14% of monocytes without LPS and 30% with LPS. MHCII expression was only detected in cells (approx. 17%) treated with LPS. CD11c and Ly6C were not detected in any isolated cells following incubation in the absence or presence of LPS (data not shown).

In addition to cell surface expression, we also observed that cells were capable of phagocytosing a range of substances over a 24 h incubation period. Specifically, CD11b-positive cells isolated from 6-month-old mice appeared the most effective phagocytes of FITC-Aβ_1–42_ (**Fig. 2I, Fig. 3C & 3D**), where approximately 23% of the cell population were positive for or able to take up this protein compared to 16% that could phagocytose 1 μm fluorescent microspheres (**Fig. 2G**) and 6% that were capable of phagocytosing FITC-Dextran (**Fig. 2H**; **Table 1**). Here, we also observed that LPS could significantly enhance phagocytosis of FITC-Aβ_1–42_ (42%, p < 0.01) and the 1 μm microspheres (25%, p < 0.05), however, had no effect on the uptake of FITC-Dextran (4%; **Table 1**). Surprisingly, we found that young monocytes less effectively phagocyted FITC-Aβ_1–42_ (3%) compared to monocytes isolated from 6-month-old mice. This was also true for the phagocytosis of FITC-Dextran, however, young monocytes displayed similar uptake levels for 1 μm microbeads (14%) compared to adult monocytes. We also observed that the phagocytosis of FITC-Aβ_1–42_ (17%) and FITC-Dextran (4%) by young cells was significantly enhanced by LPS treatment (p < 0.01; **Table 1**).

**Table 1:** Evaluating phagocytosis in peripheral blood CD11b-positive mouse monocytes.

**Substance**	**Adult monocytes**		**Young monocytes**	
	**(-)**	**+ LPS**	**(-)**	**+ LPS**
1 μm microbeads	16.0 ± 3.0 (8)	25.2 ± 3.1 (5) *	14.4 ± 1.9 (11)	16.8 ± 1.8 (10)
FITC-Dextran	6.0 ± 1.1 (9)	4.3 ± 1.9 (6)	0.3 ± 0.1 (11)	3.9 ± 0.8 (9) **
FITC-Aβ_1–42_	22.7 ± 6.6 (8)	42.1 ± 7.6 (8) **	2.9 ± 0.5 (13)	16.5 ± 2.8 (9) **

CD11b+ mouse monocytes were isolated from adult (6 months) or young (14 days) mice (C57BL/6N) using the Pluriselect pluriBead cell separation kit. To evaluate effective phagocytosis, approx. 5 x 10^5^ cells were incubated with various substances in the absence or presence of LPS (1 μg/ml) for 24 h at 37°C/5% CO_2._ Values are given as mean ± SEM of the percentage (%) of live cells positive for the given substance (i.e. % of cells that phagocytosed the substance). The number in parenthesis indicates the number of independent experiments. Statistical analysis was performed using a Student’s t-test comparing the % of positive cells alone to those treated with LPS (* p < 0.05, ** p < 0.01).

### Cognitive functions (learning and anxiety)

**Fig 4:**
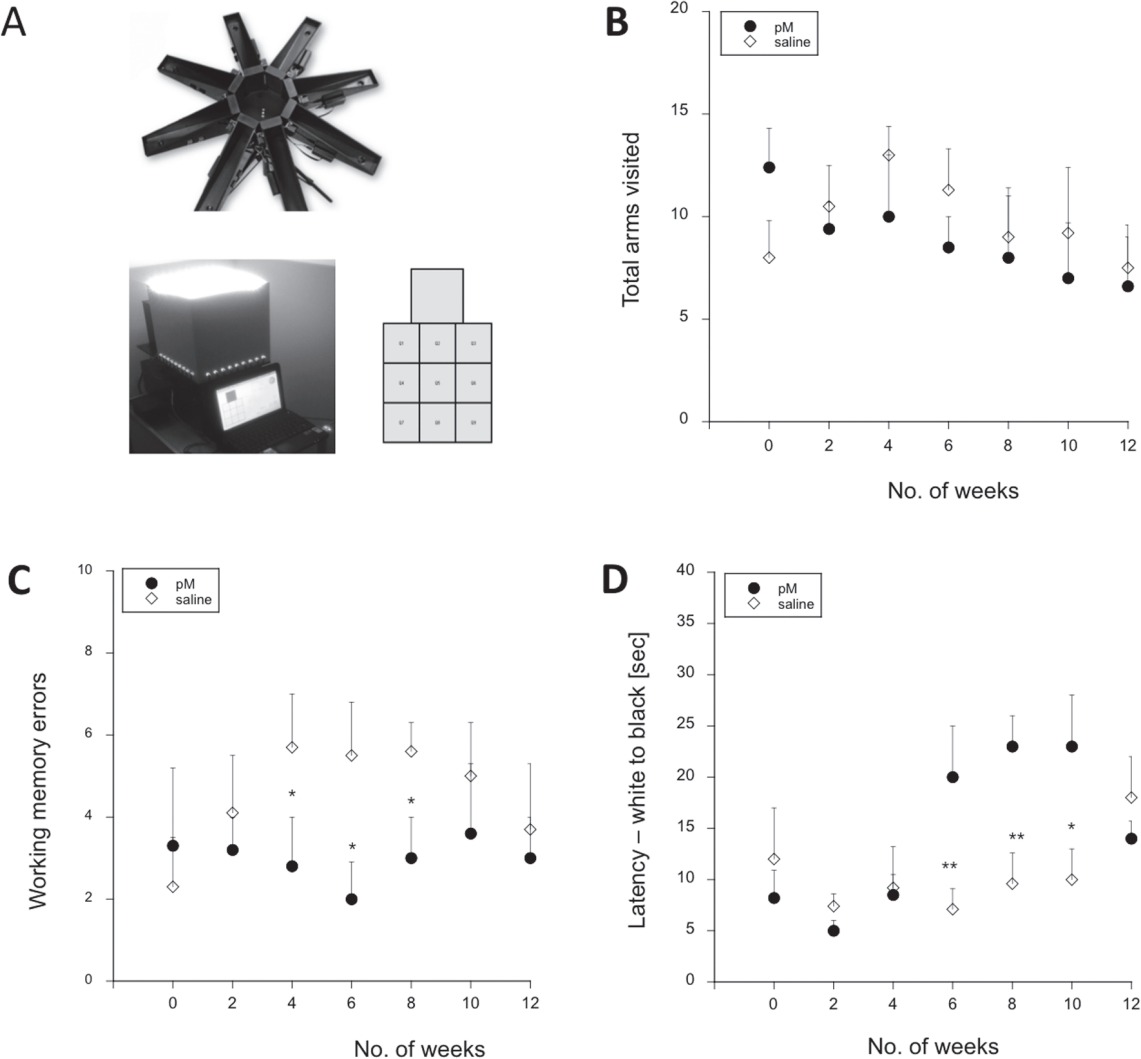
Cognitive function in monocyte-infused APPSwDI mice. Spatial learning and memory were assessed using a modified win-shift procedure in an 8-arm maze (**A**) every two weeks. Intravenous infusions of 5 x 10^6^ young monocytes (pM, filled circle; n = 8) or saline (unfilled diamond; n = 7) took place at 0, 4, and 8 weeks. To test memory function, animals were evaluated for total number of visits (**B**) to radial arms, and working memory errors (**C)** measured over two sessions. Anxiety was tested using a black/white box (**A**) over 5 sessions and quantified by the number of seconds it took the animal to leave the white area (**D**). Plotted data represents the mean ± SEM performance of each animal group (n = 5). Statistical analysis was performed using a Student’s t-test (* p < 0.05, ** p < 0.01).

In the present study, we observed that infusion of young monocytes significantly altered memory function as seen by significant reductions in working memory errors (revisits to already-visited arms). Specifically, animals exhibited a reduction in the number of working memory errors at 4 (p < 0.05), 6 (p < 0.05), and 8 (p<0.05) weeks between the 2^nd^ and 3^rd^ monocyte infusions (**Fig. 4C**). Interestingly, at similar time points animals also displayed a significant elevation in time spent in the white area of the black/white test box at 6 (p < 0.01), 8 (p < 0.01), and 10 (p < 0.05) weeks, again following the 2^nd^ and 3^rd^ monocyte infusions (**Fig. 4D**). An increase in time before leaving the white compartment suggests that animals receiving monocyte infusions were less exploratory compared to saline-infused animals, an indication for anxiety. We also showed that the number of total arms visited did not change in response to infusion (**Fig. 4B**), indicating that motor function was similar between animal groups.

### Aβ plaques and APP in the cortex

**Fig 5:**
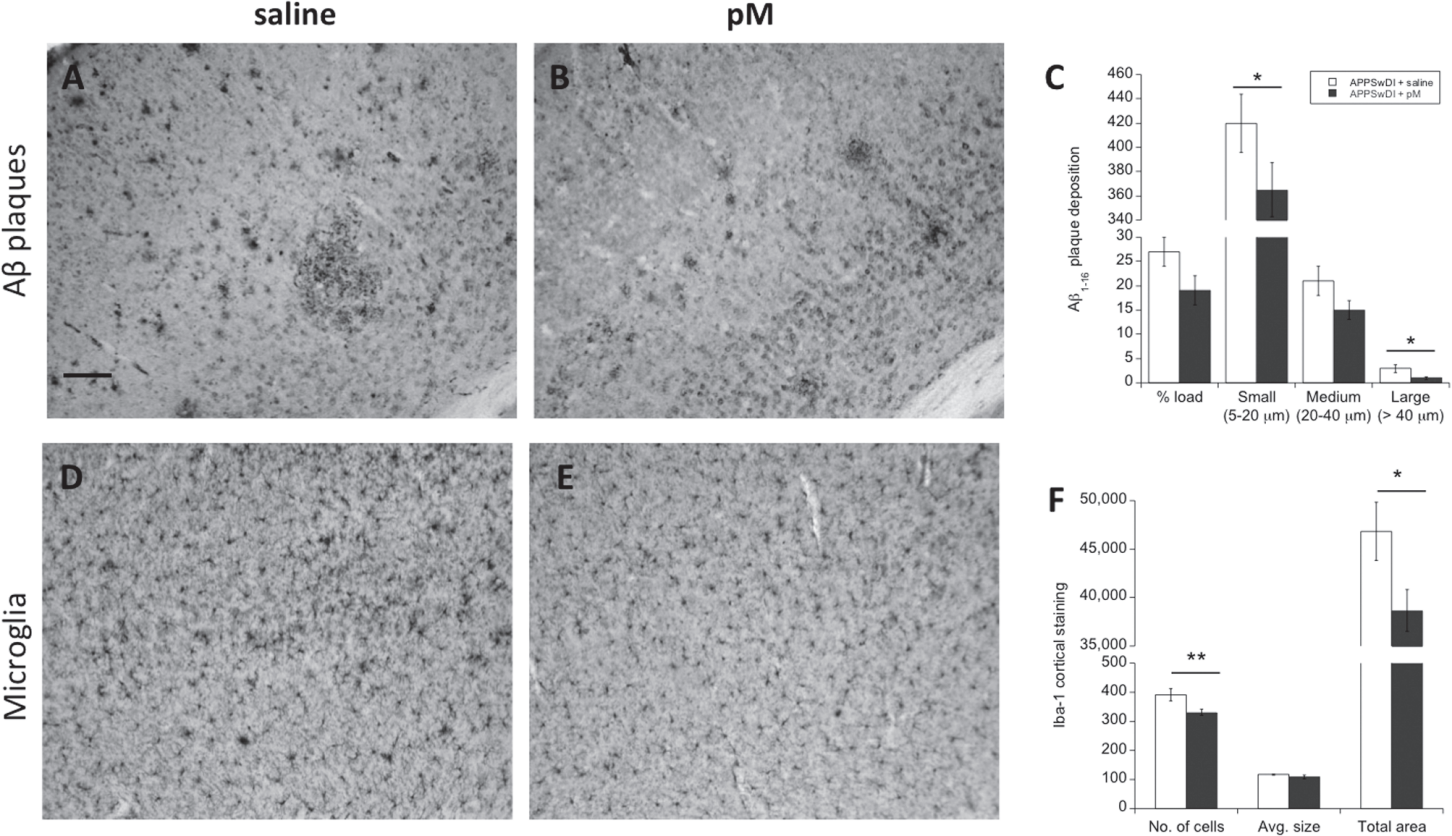
AD-like pathologies in monocyte-infused APPSwDI mice. Following monocyte infusions, brains were collected and cortical regions were evaluated for Aβ deposition (**A-C**), and microglial Iba1+ cell staining (**D-F**). APPSwDI mice receiving young peripheral blood monocyte (pM) i.v. infusions (**B,E**) are indicated with a shaded gray bar (n = 8). APPSwDI mice receiving saline (**A,D**) served as negative controls and are indicated with a white bar (n = 7). Amyloid plaque deposition was assessed using an antibody against Aβ_1–16_ for percent plaque load and specific plaque sizes in the cortex (**A-C**). Microglial cell activation was evaluated using an antibody against ionized calcium-binding adapter molecule 1 (Iba1). Bar graphs display the mean ± SEM (error bars) of plaque/axon/cell quantification. Two to four cortical sections were quantified per brain per animal. Statistical analysis was performed using a Student’s t-test (* p < 0.05, ** p < 0.01). Scale bar = 120 μm.

**Fig 6:**
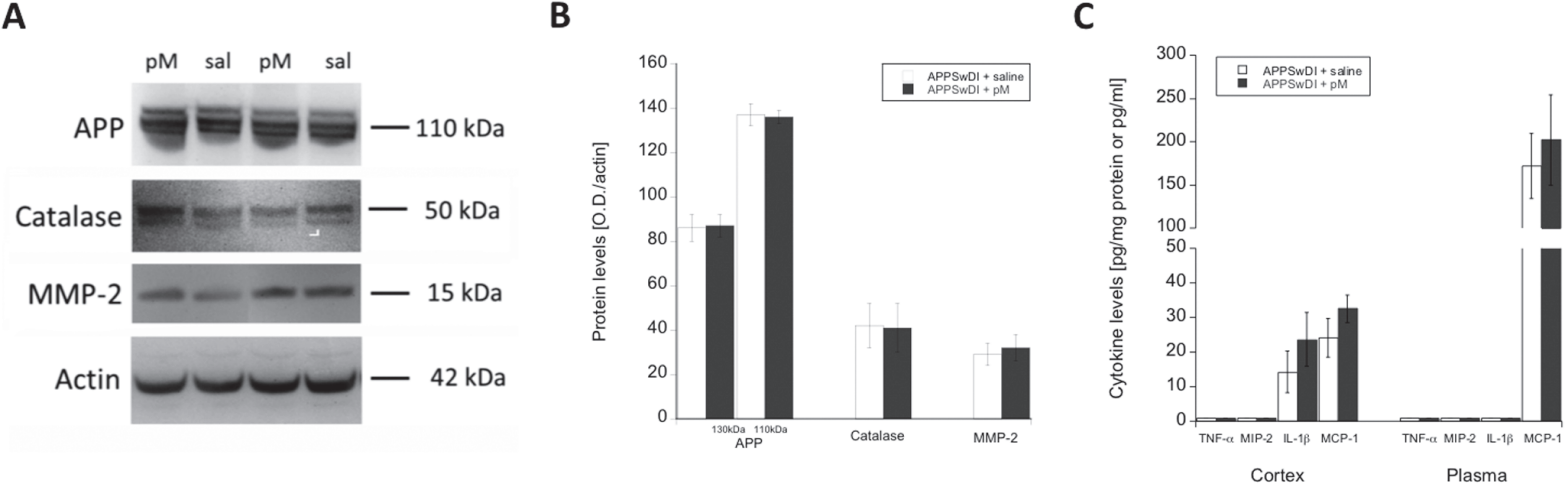
Protein levels of APP, catalase, and cytokines in monocyte-infused APPSwDI mice. Following monocyte infusions, brains were collected and cortical extracts were evaluated for amyloid precursor protein (APP), catalase, or matrix metalloproteinase-2 (MMP-2) by Western blot (**A&B**; n = 4–6 per animal group). In addition, cortex (n = 6 per animal group) and plasma (n = 4 per animal group) were evaluated for tumor necrosis factor-α (TNF-α), macrophage inflammatory protein-2 (MIP-2), interleukin-1β (IL-1β), and monocyte chemotactic protein-1 (MCP-1) levels by ELISA (**C**). APPSwDI mice receiving young peripheral blood monocyte (pM) i.v. infusions are indicated with a shaded gray bar. APPSwDI mice receiving saline (sal) served as negative controls and are indicated with a white bar. Bar graphs display the mean ± SEM (error bars) of protein levels. Statistical analysis was performed using a Student’s t-test (all not significant).

At 8 months, the brains of saline-infused (**Fig. 5A**) and monocyte-infused (**Fig. 5B**) animals were collected and evaluated for changes in Aβ deposition in different areas of the cortex and hippocampus. Here, we found that administration of young monocytes resulted in a 30% reduction in plaque load (from 27% to 19%; **Fig. 5C**). Although, this was not a statistically significant reduction, we later discovered that animals receiving monocytes showed significantly reduced numbers of size-specific Aβ plaques. In particular, our data showed that monocyte intravenous infusions resulted in a significant reduction in the number of small (i.e. 5–20 μm in diameter plaques; p < 0.05) and large plaques (i.e. >40 μm plaques; p < 0.05) (**Fig. 5C**). The total protein expression of APP (130 and 110 kDa) in the cortex was not altered as shown by Western Blots (**Fig. 6**).

### Microglia and inflammatory markers

Microglial cells were evaluated in the cortex by staining for ionized calcium-binding adapter molecule 1 (Iba1), a marker that is specifically expressed by microglia/macrophages in the brain. In particular, Iba1 expression was upregulated when these cells became activated. Here, we showed that the average number of cells expressing Iba1 in the cortex was significantly reduced (p < 0.01) in animals receiving monocyte infusions (**Fig. 5E**) compared to controls (**Fig. 5D & 5F**). This was also true for the total area (p < 0.05) of Iba1 expression in the cortex, however, we did not observe any significant changes in the cell size of Iba1-positive cells (**Fig. 5F**). Changes in microglial cell morphology were also apparent; animals receiving monocyte infusions appeared to exhibit smaller somas, indicating less cell activation, compared to those receiving only saline infusions. We did not observe any significant changes in protein expression levels of catalase, matrix metallopeptidase 2 (MMP-2), interleukin-1β (IL-1β), or monocyte chemotactic protein 1 (MCP-1/CCL-2) in the cortex between animals groups (**Fig. 6A-C**). We also evaluated cytokine levels in the plasma of these animals, however, again did not observe any significant differences in cytokine levels between animals receiving monocyte infusions and controls (**Fig. 6C**).

## DISCUSSION

In the present study, we assessed the effects of monthly administration of ‘young’ peripheral monocytes on spatial learning and memory, Aβ plaque load, microglia and inflammation. This study demonstrates that i.v. infusion of two-week-old monocytes can transiently improve cognitive function as well as reduce AD-related neuropathologies in APPSwDI adult mice.

### Monocytes and age-dependent functional alterations

Monocytes play an important role in immune responses [34]. Following their generation in the bone marrow, these cells are released into the bloodstream before migrating into peripheral organs and replenishing tissue-specific macrophage and dendritic cell populations [35, 36]. During an immune response, monocytes are recruited to sites of inflammation and/or injury and aid in further propagating an effective immune response. This is accomplished by secreting inflammatory cytokines or chemokines, differentiating into macrophages and dendritic cells or performing antigen-presenting cell activity themselves [37]. Recent investigations indicate that functional impairment in cells of the monocytic lineage may be attributed to cognitive decline and plaque accumulation in AD [4–6,11,38]. Thus, we hypothesize that replenishing these cell populations with younger and possibly better functioning monocytes could help halt cognitive impairment and the spread of AD neuropathology, exploiting their natural ability to migrate to sites of CNS injury and amyloid deposition [7,39].

Monocytes exist as diverse heterogeneous cell subsets, varying in function, based on their differential expression of cell surface markers [40,41]. Blood-derived mouse monocytes are identified by their expression of CD11b (membrane-activating complex 1, Mac-1), the F4/80 antigen, and CD115 (macrophage-colony stimulating factor, M-CSF receptor) [42]. CD11b is a specific monocytic antigen and in combination with CD45 has previously been used to distinguish activated peripheral mononuclear cells from resident macrophages/microglia [9]. A previous study has shown that CD11b-positive bone marrow-derived cells are capable of infiltrating the AD brain and reducing plaque burden [7]. Thus, we hypothesize that CD11b+ ‘young’ monocytes possess high capacity for migration to β-amyloid plaques and phagocytic activity. For ‘young’ monocytes, we used a novel CD11b isolation kit to selectively capture two-week-old cells positive for this surface marker. This age of monocyte was chosen due to their very early maturation state and possible early stem cell- or precursor-like tendencies (i.e. higher potential to differentiate into macrophages). Previous studies indicate that chronic inflammation could play a role in AD and that older monocytes become chronically activated (e.g. increasing expression of cell surface markers with age) propagating further damage and insult. Thus, we also reasoned that young two-week-old monocytes would exhibit the least amount of chronic inflammatory potential. Unfortunately, CD11b pluriBead isolation of monocytes resulted in the transient loss of all measured cell surface markers. However, following 24 h cultivation at 37°C with or without LPS, surface marker expression was restored, including cell surface markers CD11b, CD45, CD68, F4/80, and MHCII (only upon LPS stimulation).

Further, we also assessed whether the isolated CD11b cells could be effective phagocytes for Aβ and whether this uptake was specific for Aβ. Our data indicate that young monocytes can phagocytose FITC-Aβ, however, require external inflammatory stimulation (i.e. LPS) in order to do so. We also show that these cells are not as effective phagocytes of FITC-Aβ compared to 6-month-old adult monocytes. Two-week-old monocytes may not be an optimal age for functional phagocytosis. Future studies should include testing other younger age subsets (i.e. four-week-old monocytes) to determine the optimal age for monocyte phagocytosis of Aβ. It could also be possible that in vitro conditions were not conducive to promoting phagocytosis of FITC-Aβ in young cells, that FITC-Aβ is a suboptimal substrate for young cell uptake, or that further development of young cell subsets is required for more optimized phagocytosis-related mechanisms. Previous studies involving the effects of aging on phagocytosis have produced mixed results [43]. On the other hand, a recent study demonstrated that phagocytosis remained unchanged in bone marrow-derived monocytes/macrophages of aged and young mice [44], indicating that age does not alter phagocytosis. However, others have reported that phagocytosis of apoptotic cells is reduced or impaired in both aged mouse macrophages and human monocyte-derived cells [43].

### The effects of young peripheral monocyte infusions on cognitive function

APPSwDI mice typically display Aβ plaques as early as 5 months and cognitive function, as measured in an 8-arm maze, begins to significantly decline around this same time. Thus, we reasoned that if young monocytes could elicit beneficial responses against plaque accumulation and/or cognitive decline, 5 months of age would be a good point to begin administering these cells. Here, we show that monthly i.v. infusions of young two-week-old monocytes results in a transient improvement in spatial learning and memory, as seen by a significant reduction in working memory errors. It remains unclear why these effects are only transient, however, it could be possible that monthly monocyte infusions are too infrequent to result in a cumulative and constant effect on cognitive function. It could also be possible that once monocytes infiltrate the CNS and alter brain function, downregulatory processes become activated halting further change. We also observed a transient increase in anxiety-like behavior in monocyte-infused mice during this similar time period. In line with our findings, a recent investigation has demonstrated that under stress conditions bone marrow-derived monocytes are recruited into the brain and promote anxiety-like behavior [45]. Taken together, these findings indicate that infusion of peripheral monocytes in APPSwDI mice can result in transient changes to memory- and anxiety-related behavior, however, further investigations are needed to identify the underlying mechanisms.

### The effects of young peripheral monocyte infusions on beta-amyloid and APP

Accumulating evidence indicates that functional disruptions in Aβ clearance mechanisms by monocyte-derived cells or microglia may play a central role in the pathology and progression of AD. Studies have shown that microglia and macrophages isolated from AD patients exhibit impairment in phagocytosis-related mechanisms or are ineffective at phagocytosing Aβ [20,46]. Furthermore, several investigations have shown that alterations and functional impairments in monocytes and microglia correlate or coincide with cognitive decline and amyloid deposition in AD transgenic mice [47,48]. These findings indicate that a beneficial therapeutic strategy against AD could involve replenishing dysfunctional phagocytes with younger functional cells.

In the present study, we show that i.v. infusion of young monocytes does not have a significant effect on overall plaque load, however, does result in significant reductions of specific plaque sizes. These data suggest that young monocytes are able to gain entry into the brain or activate other pathways that result in plaque remodeling and possible clearance in the brain. However, it could be possible that monocyte entry into the brain is somewhat diminished due to the loss of certain surface markers immediately following bead isolation. However, we have shown that this expression reappears after 24 h, however, such findings could explain why we observe less positive effects on plaque deposition and cognition. In addition, it could be possible that monthly infusions of 5 x 10^6^ monocytes might not be frequent enough to induce a significant reduction in amyloid plaque load. A previous study demonstrated that twice weekly infusions of CD11b+ bone marrow cells (transfected with a form of the protease neprilysin) could arrest amyloid deposition in an AD transgenic mouse model [7]. Thus, more frequent infusions may be required to attenuate amyloid deposition or a longer experimental set-up that provides more time for cells to rid the brain of amyloid. Others have shown that bone marrow-derived cells (2 x 10^7^ cells injected via the tail vein in combination with chemotherapy treatment) are detected in mice as soon as 24 h and up to four weeks following hypoxic-ischemic brain injury despite injection 6 months prior [39].

Further, we were interested in determining whether these alterations also translated into changes in cortical APP protein levels. Here, we report that no significant differences in APP expression were found in the cortex between APPSwDI mice given monocyte or saline infusions. This data suggests that monocyte infusions do not alter APP processing or expression in the cortex, however, it could also be possible that changes in APP protein levels are present at earlier stages of the disease.

### The effects of young monocyte infusions on microglia and inflammation

Microglia are considered the resident immune cells of the CNS, responsible for surveying the brain and responding to first signs of insult or injury [49,50]. In both human AD and mouse transgenic AD models, activated microglia are found closely associated to amyloid plaques, indicating their potentially important role in Aβ and plaque pathology [49,51]. However, delineating the role of these cells in AD has proven difficult [11,17]. In vitro and in vivo studies have shown that microglia can phagocytose Aβ [17,52,53], whereas, others have demonstrated that complete ablation of these cells has little to no effect on plaque deposition [17,54]. Moreover, studies indicate that microglia might develop a proinflammatory or neurotoxic phenotype in response to Aβ exposure, contributing to further disease aggravation and progression [11,50]. Here, we show that Iba1 staining, a marker for microglial/macrophage activation, is significantly reduced in animals receiving monocyte infusions. The observable morphological changes (i.e. decreased size in soma) of these cells also points to diminished cell activation. These findings indicate that monocyte infusions could play a role in suppressing microglial activation. However, further investigations are needed to understand whether this also includes altering properties involved in phagocytosis, the release of neurotoxic species, and the release of proinflammatory cytokines. In addition to microglial activation, we were also interested in determining whether monocyte infusions could have a detrimental effect on cytotoxicity and neuroinflammation in the APPSwDI mouse brain. In this study, we demonstrated that monthly i.v. monocyte infusions do not significantly alter catalase, MMP-2 or proinflammatory cytokines in the cortices or plasma of APPSwDI mice. Together, these findings suggest that monocyte infusion does not propagate or aggravate neurodegeneration or neuroinflammation in AD transgenic mice, as measured by these markers. It still remains unclear why we only found subtle or transient positive effects of monocyte infusion on the parameters measured in this study. Since inflammatory mediator expression was not measured at the cellular level in microglial or neuronal cells, it could be possible that alterations in these markers at the cellular level may be responsible for the subtle changes we observed in plaque load and cognitive properties. We also did not see any changes in MCP-1 at the cortical level. Thus, we cannot rule out the possibility that monocyte infiltration did not occur under these conditions and may be the reason why we only observed subtle changes in plaque load and cognitive outcome. However, it could be possible that monocyte infiltration was not necessary to activate pathways involved in plaque alterations, and cognitive function.

To our knowledge, this study presents the first findings on the effects of i.v. infusion of young peripheral monocytes in AD transgenic mice. Our data indicates that the infusion of young monocytes induces transient, but beneficial effects on cognitive function. We also show that this treatment results in a significant reduction of AD-related neuropathology including plaque deposition and microglial activation, without leading to dramatic proinflammatory responses. Taken together, these findings provide evidence that young monocytic cells are protective against AD progression and may serve as optimal targets for AD therapeutic strategies.
